# Antibody-drug conjugates therapy for triple-negative breast cancer: a systematic review and meta-analysis

**DOI:** 10.3389/fmed.2026.1975216

**Published:** 2026-10-26

**Authors:** Ke Wan, Mengxin Liu

**Affiliations:** 1 Department of Radiology, The First Affiliated Hospital of Zhejiang Chinese Medical University (Zhejiang Provincial Hospital of Chinese Medicine), Hangzhou, China; 2 The First Clinical Medical College of Zhejiang Chinese Medical University, Hangzhou, China

**Keywords:** antibody-drug conjugates, chemotherapy, meta-analysis, safety, triple-negative breast cancer

## Abstract

**Purpose:**

This systematic review and meta-analysis aimed to compare the efficacy and safety of antibody-drug conjugates (ADCs) vs. conventional chemotherapy in patients with triple-negative breast cancer (TNBC) and to provide evidence for clinical decision-making and guideline development.

**Methods:**

We systematically searched PubMed, Embase, and Cochrane Library and extracted data including hazard ratio (HR) and 95% confidence interval (CI) for progression-free survival (PFS) and overall survival (OS), odds ratio (OR) for safety, and objective response rate (ORR).

**Results:**

This study ultimately included 6 articles (5 dedicated RCTs for TNBC and 1 exploratory analysis of an HR-negative/HER2-low subgroup from the DESTINY-Breast04 trial), including 2,479 patients (1,256 patients in ADCs treatment and 1,223 patients in conventional chemotherapy). *HR* for PFS was 0.48 [95% *CI* (0.38, 0.61), *P* = 0.001], and *HR* for OS was 0.60 [95% *CI* (0.46, 0.79), *P* = 0.027]. *OR* for incidence of all adverse events (AEs) was 1.05 [95% *CI* (0.86,1.29), *P* = 0.901], and *OR* for incidence of grade ≥ 3 AEs was 1.11 [95% *CI* (0.88, 1.42), *P* = 0.984]. The pooled results demonstrated that ADCs significantly improved OS and PFS, while showing no significant increase in overall AEs or grade ≥ 3 AEs compared to control.

**Conclusion:**

This study suggests that ADCs significantly improve OS and PFS without significantly increasing grade ≥ 3 AEs compared to conventional chemotherapy in TNBC, providing support for clinical decision-making and guideline development. These findings may inform future head-to-head trials, optimization of ADC-based combination regimens, and biomarker-guided precision treatment. As clinical data on novel ADCs continue to accumulate, these conclusions may be further validated and extended.

## Introduction

Breast cancer is the most common malignant tumor among women worldwide and the second leading cause of cancer-related death. Breast cancer mortality has decreased by approximately 44% since 1989; however, prognosis varies substantially among molecular subtypes (1, 2). Triple-negative breast cancer (TNBC), defined by the absence of estrogen receptor (ER), progesterone receptor (PR), and human epidermal growth factor receptor 2 (HER2) expression, accounts for approximately 15%–20% of breast cancers and is associated with a high risk of recurrence and limited treatment options (3, 4).

Due to the lack of relevant receptor markers, patients with TNBC cannot benefit from established endocrine or HER2-targeted drugs, and there is currently no effective specific targeted therapy for TNBC (5). Therefore, the standard treatment for TNBC is biomarker-guided systemic therapy, including chemotherapy, immunotherapy, ADCs, and PARP inhibitors (6). However, TNBC is biologically aggressive. Although TNBC is sensitive to conventional chemotherapy regimens (the synergistic use of platinum drugs with taxanes or anthracyclines), its use is limited by high toxicity, non-selective tumor targeting, and gradually reduced efficacy with increasing lines of treatment, resulting in a still poor prognosis (7). In addition, TNBC is characterized by a higher rate of recurrence and distant metastasis, resulting in a lower survival rate. Less than 30% of TNBC patients achieve a clinical complete response, and nearly 40% of stage I to III TNBC patients experience disease recurrence within 2 to 3 years after conventional treatment (8, 9). Recurrence and mortality rates remain substantially higher in TNBC than in non-TNBC subtypes.

Antibody-drug conjugates (ADCs) are composed of a tumor-associated antigen-targeting monoclonal antibody, a cytotoxic drug, and a chemical linker that connects the two (10). These drugs deliver cytotoxic agents precisely to cancer cells through the antibody component, thereby enhancing therapeutic efficacy and reducing damage to normal tissues (11). The DREAMM-7 study showed that Belantamab mafodotin plus bortezomib and dexamethasone for refractory multiple myeloma had a hazard ratio (*HR*) for overall survival (OS) of 0.58 [95% confidence interval (*CI*) 0.43–0.79; *P* = 0.0002] compared with the control group (13); A meta-analysis by Plattel showed that Brentuximab vedotin monotherapy can effectively improve the prognosis of patients with refractory Hodgkin lymphoma. Among patients who completed four cycles, the objective response rate (ORR) reached 62.6%, 95% *CI* (56.0%–68.9%), and the complete response rate was 32.9%, 95% *CI* (20.8%–46.3%). Long-term survival data show that the 1-year, 2-year and 5-year progression-free survival (PFS) rates are 52.1%–63.2%, 45.2%–56.2% and 31.9%–33.0% respectively; the corresponding OS rates are 68.2%–82.7%, 58.0%–81.9% and 58.0%–62.0% (14), demonstrating the advantages of ADCs in the field of hematologic tumors. In solid tumor therapy, ADCs have shown considerable potential, particularly for the treatment of TNBC (12, 15, 16). At present, ADCs have become a new therapeutic category in breast cancer, and the U.S. Food and Drug Administration (FDA) has approved drugs such as Datopotamab deruxtecan (Dato-DXd) and Sacituzumab govitecan (SG) as standard treatment regimens for TNBC (17). Phase III studies that facilitated regulatory approval include ASCENT (19) and TROPION-Breast02 (20), demonstrated significant improvements in PFS and OS compared with chemotherapy in the relevant subgroups, with *HR* for PFS ranging from 0.41 to 0.57 and *HR* for OS ranging from 0.51 to 0.79, thereby altering clinical guidelines (16). In the TROPION-Breast02 trial (20), TNBC patients treated with Dato-DXd had a PFS *HR* of 0.57 (*P* < 0.001) and an OS *HR* of 0.79 (*P* = 0.029) compared to the chemotherapy group. Similarly, in the OptiTROP-Breast01 trial (21), TNBC patients treated with Sac-TMT had a *HR* for PFS of 0.31 (*P* < 0.001) and an OS *HR* of 0.53 (*P* < 0.001) compared with the chemotherapy group. Despite these advances, challenges remain in maximizing tumor delivery and minimizing off-target toxicity. Recent advances in nanocarrier-based targeted delivery platforms offer promising strategies to enhance tumor accumulation and reduce off-target toxicity of ADCs (31).

Although several phase III trials have evaluated the efficacy and safety of ADCs in TNBC, these trials differ in patient populations, drug types, control regimens and outcome definitions. To date, no meta-analysis has comprehensively synthesized the evidence or quantitatively compared the overall benefit–risk profiles of individual ADCs vs. conventional chemotherapy.

To this end, this systematic review and meta-analysis aimed to compare the efficacy (PFS, OS, and ORR) and safety [overall incidence of adverse events (AEs) and incidence of grade ≥3 AEs] of ADCs vs. conventional chemotherapy in patients with TNBC and to provide higher-level evidence for clinical decision-making and guideline development.

## Methods

### Study design

The meta-analysis of this study has been registered with PROSPERO (https://www.crd.york.ac.uk/PROSPERO), registration number is (CRD420261452989). This study adhered to the Preferred Reporting Items for Systematic Reviews and Meta-Analyses (PRISMA 2020) guidelines (27). The inclusion criteria for this study are: 1. Type of study: Randomized controlled trial (RCT); 2. Study subjects: Patients with pathologically confirmed locally advanced or metastatic triple-negative breast cancer; 3. Intervention: The experimental group received ADCs monotherapy (18–22) or combination therapy (23); 4. Control group: receiving conventional chemotherapy; 5. Outcome indicators: At least one of the following indicators should be reported: OS, PFS, ORR, safety. Exclusion criteria: 1. Non-original research (reviews, meta-analyses, case reports, commentaries, letters, etc.); 2. Animal experiments or *in vitro* studies; 3. Literature that is repeatedly published or has incomplete data that cannot be extracted; 4. Non-English literature. The data were independently extracted by two researchers. The general study characteristics included first author, year of publication, country, study design, sample size, detailed neoadjuvant chemotherapy (NAC) regimen, age, and follow-up time; outcome indicators: *HR* and 95% *CI* of OS, *HR* and 95% *CI* of PFS, ORR, and incidence of AEs. Any discrepancies that arise during screening or data extraction are addressed through consultation with another researcher.

### Search strategy

The study selection process is presented in Figure 1. This study was conducted in accordance with the PRISMA guidelines for systematic reviews and meta-analyses. We performed a systematic search of the PubMed, Embase, and Cochrane Library electronic databases on April 15, 2026. The search strategy combined subject terms and keywords, including “Antibody Drug Conjugate*”, “ADC”, “sacituzumab govitecan”, “Triple Negative Breast Cancer”, “TNBC”, etc. The full search strategy is provided in Supplementary Material 1. In addition, this study also reviewed the abstracts and reports of major international conferences from 2019 to 2026 (the Annual Meeting of the American Association for Cancer Research, the Annual Meeting of the American Society of Clinical Oncology, the European Society for Medical Oncology Congress, the World Conference on Lung Cancer, and the European Lung Cancer Conference), and examined the reference lists of the latest relevant reviews and meta-analyses to ensure the completeness of references. All studies identified from the search underwent a two-stage screening process: first, a preliminary screening of titles and abstracts was conducted, with 460 articles passing the initial screening. Subsequently, the full texts of the screened studies were reviewed, and 454 were excluded after full-text screening. A two-stage screening process was applied to all retrieved studies. First, titles and abstracts were screened, yielding 460 articles for full-text review. After full-text assessment, 454 articles were excluded (21 articles had no extractable data, 332 were single-arm studies, and 101 did not report any of the study endpoints required for this research) and 6 articles (5 dedicated RCTs for TNBC and 1 exploratory analysis of an HR-negative/HER2-low subgroup from the DESTINY-Breast04 trial) (18–23) were ultimately included in the final analysis. Two authors of this study independently assessed study eligibility against the inclusion criteria.

**Figure 1:**
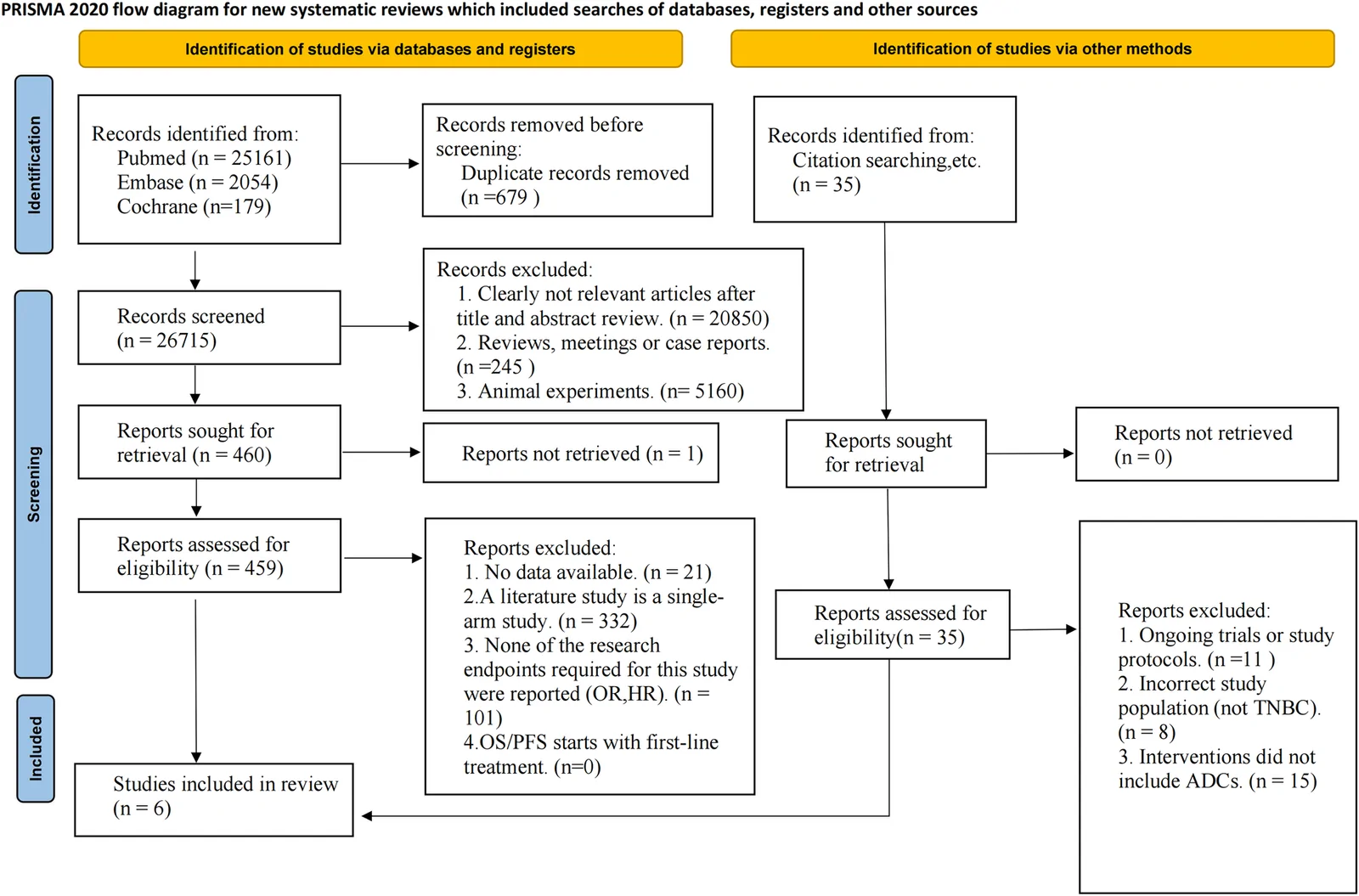
PRISMA flow chart for the included studies. On April 15, 2026, we conducted a systematic search of the Pubmed, Embase, and Cochrane electronic databases. We initially found 27,394 articles. After removing 679 duplicates, 26,715 remained. We excluded 26,255 after title and abstract screening and 454 after full-text review, finally including 6 RCTs. PRISMA, Preferred Reporting Items for Systematic Reviews and Meta-Analyses; OR, Odds Ratio; HR, Hazard Ratio; PFS, Progression-Free Survival; OS, Overall Survival.

### Quality assessment

Two reviewers used standardized forms to extract study design, sample size, demographics, treatment protocols, follow-up, and outcomes; disagreements were resolved by consensus or third-party adjudication. Risk of bias was assessed with the Cochrane RoB 2 tool (28) across randomization, deviations from intended interventions, missing outcome data, outcome measurement, and selective reporting, with judgments of low risk, some concerns, or high risk. Across the six phase III trials, all investigations exhibited low risk of bias in all domains, reflecting robust methodology (central/randomized allocation, prespecified analyses, BICR for PFS, and objective OS). Figure 2 provides RoB 2 evaluations.

**Figure 2:**
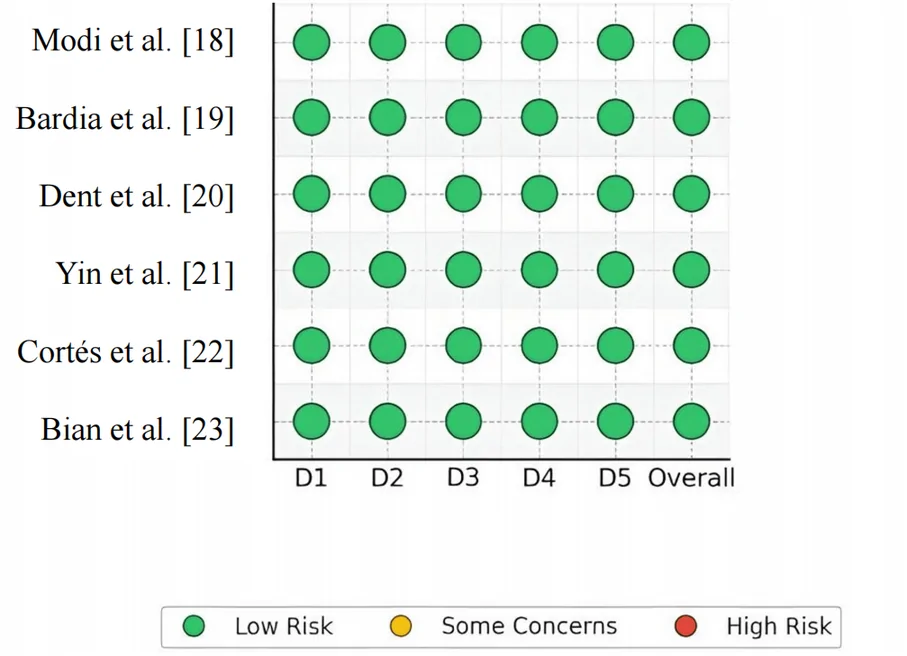
Systematic evaluation of potential bias among incorporatedstudies employing the cochrane RoB2 instrument. D1: Randomization Process | D2: Deviations from Intended Interventions | D3: Missing Outcome Data | D4: Measurement of Outcomes | D5: Selection of Reported Results.

### Statistical analysis

For binary outcomes (ORR and safety), odds ratios (*OR*s) with 95% *CI*s were calculated. For survival outcomes (OS and PFS), *HR*s with 95% *CI*s were calculated. Heterogeneity was assessed using Cochran's *Q* test (*P* < 0.10 indicating significant heterogeneity) and the *I*^2^ statistic. If *I*^2^ ≤ 50%, a fixed-effect model (Mantel-Haenszel method) was used; if *I*^2^ > 50%, a random-effects model (DerSimonian-Laird method) was applied. Publication bias was assessed using Egger's test and funnel plots. All tests were two-sided, and *P* < 0.05 was considered statistically significant. Analyses were conducted in Stata (version 18.0).

## Results

In this study, a total of 27,394 articles were initially retrieved. After removing 679 duplicate articles, 26,715 remained. Following an initial screening of titles and abstracts, 26,255 articles were excluded. After a full-text assessment, 454 articles were excluded, and 6 RCTs (18–23) involving 2,479 TNBC patients (1,256 in the ADC group and 1,223 in the control group) were ultimately included. Baseline characteristics of the included studies are summarized in Table 1. Specifically, Modi et al. (18) evaluated the Trastuzumab deruxtecan (T-DXd), Bardia et al. (19) and Cortés et al. (22) evaluated the SG, Dent et al. (20) evaluated the Dato-DXd, Yin et al. (21) evaluated the Sacituzumab tirumotecan (Sac-TMT), and Bian et al. (23) evaluated the SG combined with immunotherapy. Among them, five studies (18–22) evaluated ADCs monotherapy, and one study (23) evaluated ADC combination therapy.

**Table 1:** Basic information of the included literature.

Country	Year	First author	Total sample		Pathological type
America	2024	Bardia.et al. (19)	529	Sacituzumab govitecan (SG) 267	Second-line and beyond (≥2 prior chemotherapy lines)
				treatment of physician's choice 262	
America	2025	Bian et al. (23)	443	Sacituzumab govitecan (SG) + immunotherapy 221	First-line
				chemotherapy + immunotherapy 222	
China	2025	Yin et al. (21)	263	Sacituzumab tirumotecan (Sac-TMT) 130	Second-line and beyond (≥2 prior chemotherapy lines)
				physician's choice of chemotherapy 133	
Japan, America, the UK	2025	Dent et al. (20)	628	Datopotamab deruxtecan (Dato-DXd) 319	First-line
				investigator's choice of chemotherapy 309	
America	2025	Cortés et al. (22)	558	Sacituzumab govitecan (SG) 279	First-line
				Chemotherapy 279	
Japan, America, the UK	2025	Modi et al. (18)	58	Trastuzumab deruxtecan (T-DXd) 40	Second-line and beyond (≥2 prior chemotherapy lines)
				physician's choice of chemotherapy 18	

Efficacy analysis results: Four studies (18–21) reported OS outcomes. The meta-analysis showed that OS was significantly prolonged in the ADC group compared with the control group (*HR* = 0.60, 95% *CI*: 0.46–0.79, *P* < 0.05), with heterogeneity test *I*^2^ = 67.3%, *P* = 0.027; Six studies (18–23) reported PFS outcomes. The meta-analysis showed that PFS was significantly prolonged in the ADC group compared with the control group (*HR* = 0.48, 95% *CI*: 0.38–0.61, *P* < 0.05), with heterogeneity test *I*^2^ = 76.4%, *P* = 0.001.Five studies (19–23) reported ORR results, with the ORR of ADCs being 31%–63%, and the ORR of the control group being 4%–53%. Three studies (19, 20, 22) reported safety outcomes. Across the included studies, the incidence of AEs was 99%–100% in the ADC group and 83%–98% in the control group, while the incidence of grade ≥ 3 AEs was 33%–73% and 29%–65%, respectively. Pooled analysis showed no significant difference between ADCs and conventional chemotherapy for overall AEs (*OR* = 1.05, *P* = 0.901) or grade ≥ 3 AEs (*OR* = 1.11, *P* = 0.984). Although the point estimates were numerically higher in the ADC group, the differences were not statistically significant. However, this pooled analysis may obscure the clinically decisive differences in class-specific toxicities among individual agents. Specifically, SG is predominantly associated with hematological toxicities, particularly neutropenia (43%–52% grade ≥ 3) and diarrhea, as observed in the ASCENT and ASCENT-03 trials (19, 22). In contrast, Dato-DXd exhibits a distinct safety profile characterized by oral mucositis/stomatitis (57% any grade) and ocular surface events (24% any grade), with a lower incidence of severe hematological toxicity (20). The pooled effect sizes for PFS, OS, all AEs and grade ≥ 3 AEs were visualized in Figure 3. The scatter plots of Egger's test are displayed in Supplementary Material 2.

**Figure 3:**
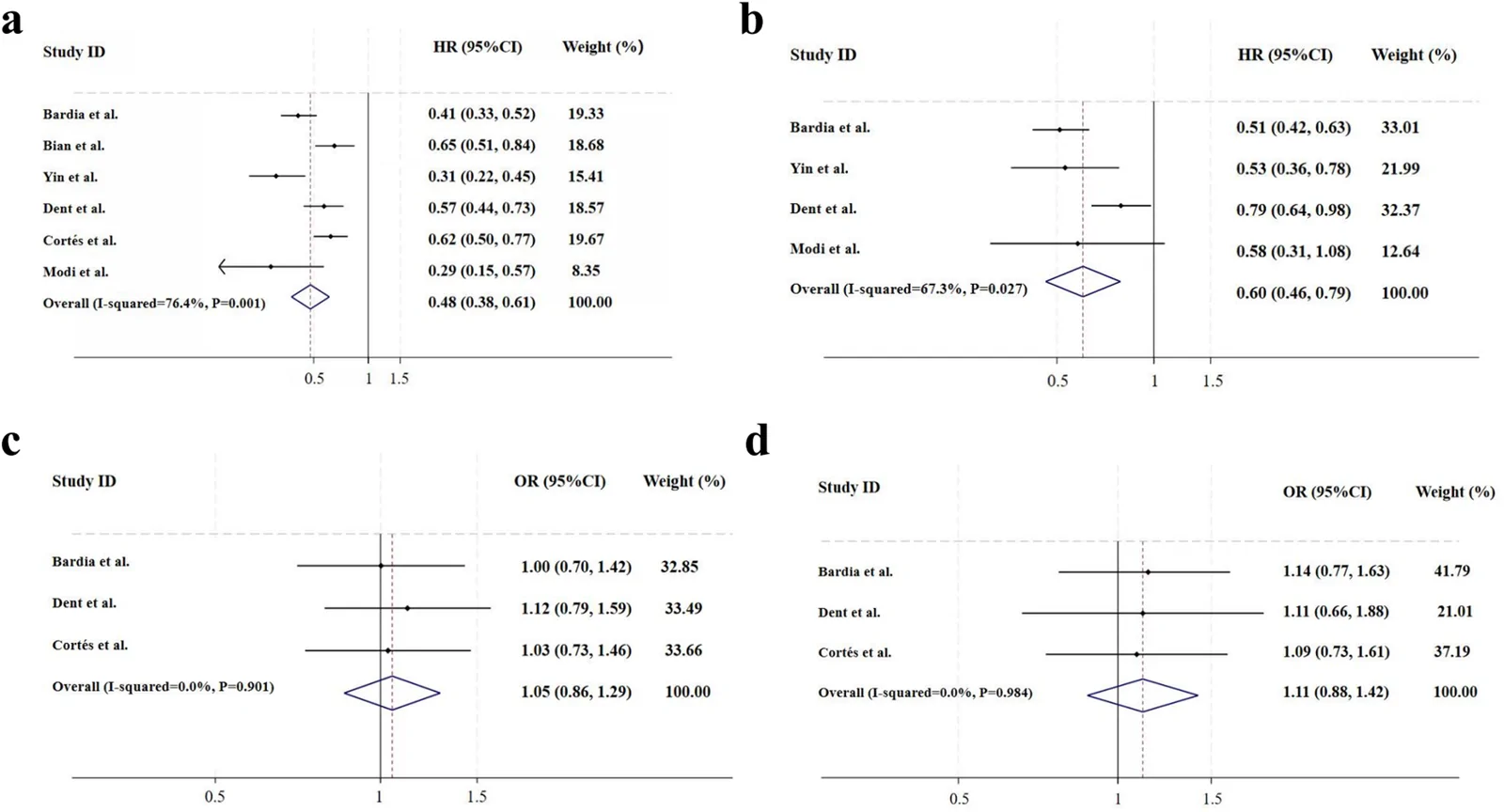
Forest plot of ADCs’ efficacy and safety. **(A)** Forest plot of progression-free survival (PFS). **(B)** Forest plot of overall survival (OS). **(C)** Forest plot of odds ratio (OR) for incidence of all adverse events (AEs). **(D)** Forest plot of OR for incidence rate of grade ≥ 3 AEs.

## Discussion

TNBC currently does not have a very good treatment plan. ADCs have already shown sufficient therapeutic potential, so we conducted a meta-analysis of RCTs directly comparing ADCs with the chemotherapy regimen chosen by doctors. We included the recently published RCTs data (18–23) in the analysis, ensuring the timeliness of the conclusions and accurately reflecting the best evidence available in current clinical practice. By integrating high-quality RCTs, the overall advantage of ADCs compared to chemotherapy in metastatic TNBC has been confirmed from the dual dimensions of efficacy and safety. This strategy has universal value that can be applied to a broad patient population in clinical practice, simplifying the decision-making process and enabling more TNBC patients to truly benefit from ADCs treatment. This study found that ADCs therapy for TNBC provides significant benefits in PFS (*HR* = 0.48, *P* = 0.001) and OS (*HR* = 0.60, *P* = 0.027).

As a core drug for precision treatment of breast cancer, ADC is covalently assembled from three parts: tumor-specific humanized monoclonal antibody, controllable cleavable linker, and highly active cytotoxic payload (10). The antibody can target and recognize breast cancer-specific membrane antigens and mediate complex endocytosis (24). The potent payload released after lysosomal degradation induces tumor apoptosis by blocking DNA replication or microtubule synthesis. Compared with conventional chemotherapy without targeted selectivity, ADCs can achieve targeted accumulation of cytotoxic drugs in breast lesions (25), with effective intratumoral drug concentrations increased by hundreds of times compared to systemic chemotherapy (26). In chemotherapy-resistant breast cancer subtypes such as HER2-low expression and triple-negative, the ORR and long-term survival benefits are significantly better than conventional chemotherapy, while substantially reducing severe myelosuppression, neurotoxicity, and gastrointestinal damage caused by chemotherapy, offering a wider therapeutic window (18, 22). Liu et al. (29) compared the cytotoxic effects of Trop-2 ADC and free topoisomerase chemotherapy drugs in breast cancer organoids and animal models, showing that the ADC had higher sensitivity in post-chemotherapy residual TNBC organoids. Cheung A et al. (30) further confirmed that EGFR-targeted ADCs exhibit higher tumor accumulation and stronger *in vivo* antitumor activity compared with free CDK chemotherapeutic agents. Future studies may also explore combining ADCs with other nanocarrier-based targeted delivery platforms to further improve tumor accumulation and reduce off-target toxicity (31).

In the included studies, the *HR* for PFS comparing ADCs with chemotherapy ranged from 0.29 to 0.65 (18–23), and the pooled *HR* value for PFS in our study was 0.48; The *HR* for OS of comparing ADCs with chemotherapy ranged from 0.51 to 0.79 (18–23), and the pooled *HR* for OS in our study was 0.60, indicating that our study results correspond to previous results. Although the pooled analysis of the six included RCTs demonstrated a statistically significant PFS benefit, Modi et al. (18) showed no statistically significant difference in OS. The main reason is that this subgroup was only a pre-specified exploratory analysis, and no separate sample size calculation was performed, with only 40 patients in the T-DXd group and 18 in the chemotherapy group, resulting in insufficient statistical power. In addition, baseline disease characteristics were imbalanced between the two groups, which may have contributed to the lack of a statistically significant difference in OS *HR*. The included populations varied across studies and comprised several clinical subgroups: patients with stage IV HER2-low metastatic breast cancer (including hormone receptor-negative disease), patients with first-line metastatic TNBC unsuitable for immunotherapy, patients with first-line PD-L1-positive TNBC, and patients with second-line or later TNBC. Despite these differences, all studies consistently confirmed the significant advantage of ADCs over chemotherapy in TNBC, but the extent of benefit varies depending on the line of treatment, with the relative benefit appearing more prominent in later lines of therapy (*HR* for PFS: 0.29–0.41 (18, 19, 21), *HR* for OS: 0.51–0.53 (19, 21). In terms of safety, this meta-analysis did not detect any statistically significant difference in the incidence of overall AEs or grade ≥ 3 AEs. These safety findings are specific to the two agents (SG and Dato-DXd), included in this meta-analysis and should not be extrapolated to other ADCs. This consistent trend across studies and populations further consolidates the therapeutic position of ADCs in metastatic TNBC and suggests that clinicians should individualize ADCs selection and combination strategies based on the patient's molecular subtype and treatment history (32). The efficacy of ADCs in hematologic tumors depends on the precise matching of the target, molecular design, and treatment strategy. Successful examples include ADCs targeting BCMA (13) or CD30 (14). Their target antigens are highly expressed on the surface of tumor cells and undergo rapid internalization, with limited expression in normal tissues, thereby contributing to high efficacy and low toxicity. In addition, cleavable linkers and potent payloads enable the elimination of tumor cells with low target antigen expression through the bystander effect, and combining these ADCs with agents such as bortezomib can synergistically enhance sensitivity (13). Some studies did not achieve the anticipated efficacy, largely due to high heterogeneity in target expression, antigen downregulation leading to resistance after prolonged treatment, or dose-limiting toxicity that narrows the therapeutic window (33). Therefore, the rational combination of an optimal target, an appropriate payload, and a reasonable regimen is the key for ADCs to achieve efficacy in hematologic tumors.

This study has several limitations. Firstly, the number of included RCTs is limited, and the total sample size is relatively small, which may affect the stability and generalizability of the pooled effect size. It is hoped that future research can expand the sample size, include more multicenter, large-sample, prospective RCTs, and further validate the combined effect results of this study. Secondly, due to the lack of individual patient data in published literature, it is not yet possible to explore in depth the modifying effects of variables such as Trop-2 expression levels and prior treatment history on efficacy. It is anticipated that subsequent research will actively collect and integrate individual patients' original clinical data, carry out targeted deep statistical analyses such as regression analysis and interaction analysis, and thoroughly explore the mechanisms by which key variables such as Trop-2 expression levels, patients' previous treatment regimens, and the number of treatments affect drug efficacy. Thirdly, in terms of safety, the standards for reporting AEs in some studies are not entirely consistent, which may introduce some bias into the pooled analysis of overall safety. Looking forward to subsequent relevant clinical studies to standardize the evaluation, recording, and reporting of AEs and to conduct standardized pooled analyses of safety outcomes. Fourthly, the inclusion of the exploratory HR-negative, HER2-low subgroup from DESTINY-Breast04 (*n* = 58) may have introduced potential bias due to its small sample size, exploratory nature, and lack of independent power calculation. Finally, owing to the limited follow-up duration in the original studies, the long-term resistance patterns and mechanisms of acquired resistance remain to be further elucidated. We look forward to subsequent studies extending the clinical follow-up period to continuously track patients' long-term treatment outcomes, disease recurrence and progression, and the occurrence of drug resistance, and to systematically characterize the patterns of long-term resistance. In addition, combining basic experiments and molecular biology approaches will be essential to further explore the molecular mechanisms underlying acquired resistance following ADCs treatment.

## Conclusion

This meta-analysis, by integrating the currently available high-quality RCTs evidence, confirms that ADCs achieve superior efficacy vs. chemotherapy with no statistically significant difference in safety, providing a solid evidence-based foundation for incorporating ADCs into preferred treatment regimens in clinical practice. This meta-analysis did not detect any statistically significant difference in the incidence of overall AEs or grade ≥ 3 AEs. At the same time, the systematically quantified safety characteristics of this study also provide data support for clinical monitoring and informed decision-making. It marks a significant advance toward more precise and effective systemic treatment strategies that improve survival and quality of life for patients with advanced disease. This study points the way for future head-to-head comparisons, optimization of combination strategies, and exploration of biomarker-driven precision therapy. As clinical data for more new ADCs continue to accumulate, this conclusion is expected to be further validated and expanded.

## Data Availability

The original contributions presented in the study are included in the article/Supplementary Material, further inquiries can be directed to the corresponding author/s.

## Supplementary material

Supplementary Material 1 Search strategies.

Supplementary Material 2 Scatter plots of Egger's test.
