# Supplementary material for: Antibody-drug conjugates therapy for triple-negative breast cancer: a systematic review and meta-analysis
Source: Front Med (Lausanne). 2026 Oct 26;13:1975216. doi: 10.3389/fmed.2026.1975216 (PMC13745321; doi:10.3389/fmed.2026.1975216)
Supplement: Supplementary file 1 [file Table1.docx]

(((Immunoconjugates [Mesh]) OR (((((((Radioimmunoconjugate* [Title/Abstract]) OR (ADC drugs [Title/Abstract])) OR (Antibody Drug Conjugate* [Title/Abstract])) OR (sacituzumab govitecan [Title/Abstract])) OR (datopotamab deruxtecan [Title/Abstract])) OR (sacituzumab tirumotecan [Title/Abstract])) OR (Immunoconjugates [Title/Abstract]))) AND (Triple Negative Breast Neoplasms [Mesh])) OR ((((((((ER Negative PR Negative HER2 Negative Breast Cancer [Title/Abstract]) OR (ER Negative PR Negative HER2 Negative Breast Neoplasms [Title/Abstract])) OR (Triple Negative Breast Cancer [Title/Abstract])) OR (TNBC [Title/Abstract])) OR (Triple Negative Breast Neoplasms [Title/Abstract])) OR (three negative breast cancer [Title/Abstract])) OR (triple-negative carcinoma [Title/Abstract])) OR (triple negative breast cancer cell line [Title/Abstract]))
